# The Impact of the COVID-19 Pandemic on Women’s Reproductive Health

**DOI:** 10.3389/fendo.2021.642755

**Published:** 2021-03-22

**Authors:** Niamh Phelan, Lucy Ann Behan, Lisa Owens

**Affiliations:** ^1^ Faculty of Health Sciences, School of Medicine, Trinity College Dublin, Dublin, Ireland; ^2^ Department of Endocrinology, St. James’s Hospital, Dublin, Ireland; ^3^ Department of Endocrinology, Tallaght University Hospital, Dublin, Ireland

**Keywords:** menstrual abnormalities, COVID-19 pandemic, psychological distress, oligomenorrhea/amenorrhea, libido, dysmenorhea, menorrhagia

## Abstract

**Background:**

The COVID-19 pandemic has profoundly affected the lives of the global population. It is known that periods of stress and psychological distress can affect women’s menstrual cycles. We therefore performed an observational study of women’s reproductive health over the course of the pandemic thus far.

**Materials and Methods:**

An anonymous digital survey was shared by the authors *via* social media in September 2020. All women of reproductive age were invited to complete the survey.

**Results:**

1031 women completed the survey. Mean age was 36.7 ± 6.6 years (range, 15–54). 693/70% reported recording their cycles using an app or diary. 233/23% were using hormonal contraception. 441/46% reported a change in their menstrual cycle since the beginning of the pandemic. 483/53% reported worsening premenstrual symptoms, 100/18% reported new menorrhagia (p = 0.003) and 173/30% new dysmenorrhea (p < 0.0001) compared to before the pandemic. 72/9% reported missed periods who not previously missed periods (p = 0.003) and the median number of missed periods was 2 (1–3). 17/21% of those who “occasionally” missed periods pre-pandemic missed periods “often” during pandemic. 467/45% reported a reduced libido. There was no change in the median cycle length (28 days) or days of bleeding (5) but there was a wider variability of cycle length (p = 0.01) and a 1 day median decrease in the minimum (p < 0.0001) and maximum (p = 0.009) cycle length. Women reported a median 2 kg increase in self-reported weight and a 30-min increase in median weekly exercise. 517/50% of women stated that their diet was worse and 232/23% that it was better than before the pandemic. 407/40% reported working more and 169/16% were working less. Women related a significant increase in low mood (p < 0.0001), poor appetite (p < 0.0001), binge eating (p < 0.0001), poor concentration (p < 0.0001), anxiety (p < 0.0001), poor sleep (p < 0.0001), loneliness (p < 0.0001) and excess alcohol use (p < 0.0001). Specific stressors reported included work stress (499/48%), difficulty accessing healthcare (254/25%), change in financial (201/19%) situation, difficulties with home schooling (191/19%) or childcare (99/10%), family or partner conflict (170/16%), family illness or bereavement (156/15%).

**Conclusions:**

The COVID-19 pandemic has significantly impacted the reproductive health of women. The long term health implications of this are yet to be determined and future studies should address this.

## Introduction

The COVID-19 pandemic, caused by severe acute respiratory syndrome coronavirus 2 (SARS-CoV-2) (1, 2), has caused over 106 million infections and 2.3 million deaths worldwide, as of February 2021, according to the  WHO COVID-19 Dashboard (3). The virus itself, as well as the measures taken to reduce its spread, have profoundly affected the lives of the global population. The pandemic has significantly impacted the mental health of many people within the population, resulting in loneliness, social isolation, financial strain, as well as the anxiety and fear of contracting the virus, and uncertainty for the future. Analysis of a national, longitudinal cohort study found that by late April 2020, mental health in the UK had deteriorated compared with before the COVID-19 pandemic (4). A US study in April 2020 found higher rates of psychological distress among adults, compared with 2018 (5). In this study the increase in psychological distress was greatest in women and young people aged 18–24 years. On the other hand, there is theoretically a group who have experienced reduced stress and improved mental well-being, for example those with financial security, those who have spent more time with their families and less time commuting since the outbreak of the pandemic.

It is known that periods of stress and psychological distress can affect women’s menstrual health. Stressors can activate the hypothalamic-pituitary-gonadal (HPG) axis and can alter the neuro-modulatory cascade that drives gonadotropin releasing hormone (GnRH) regulation (6). This can result in functional hypothalamic amenorrhoea (FHA), chronic anovulation which is not due to an underlying organic cause (7, 8). Behavioural modification such as cognitive behavioural therapy can reverse this amenorrhoea (9). FHA also occurs secondary to excessive exercise, dieting and caloric restriction and disordered eating (10, 11).

Psychological distress is not only associated with missed periods but also worsening of symptoms associated with menstruation and psychosexual health. Dysmenorrhoea has been shown to be associated with high stress levels (12), emotional instability and depression (13). Pre-menstrual symptoms (PMS) and menorrhagia are also associated with high psychological distress (14, 15). Higher perceived stress is also associated with lower libido in women (16).

Our objective therefore was to survey the general female population of reproductive age with regards to their menstrual cycle, libido and changes in their lifestyle over the course of the pandemic thus far. We performed an anonymous observational study by circulating a survey *via* social media and text message.

## Materials and Methods

### Study Design

This was an anonymous observational study. A digital survey was created using the Typeform platform (www.typeform.com). A link to the survey was shared by the authors *via* social media (Facebook, Twitter) and all women of reproductive age were invited to participate. The survey contained 50 questions on demographic information, menstrual cycle and mental health symptoms, diet, exercise and working patterns from before and since the beginning of the pandemic. It took between 5 and 10 min to complete. Ethical approval was granted for the study by the St James’s and Tallaght University Hospital Research Ethics Committee (JREC 2020-10-CA-10). Written consent was not required as all data was collected anonymously. The survey was circulated over a 2-week period in the second half of September 2020. The study was conducted and reported according to published best practice guidelines for reporting observational studies (17). A full list of survey questions can be found in **Supplementary Table 1**.

### Participants

In total 1031 women completed the survey. All women of reproductive age were invited to participate *via* digital link. Menstrual cycle and BMI data were excluded from women who became pregnant or delivered a baby during the pandemic. Menstrual cycle data was also excluded from women who stated they were amenorrhoeic for any reason (i.e. due to an intrauterine system, intrauterine device or implant, menopause, breastfeeding).

### Statistical Analysis

Data was analysed using Graphpad Prism version 8.4.3. Parametric data is reported as mean and standard deviation (SD). Non-parametric data is reported as median and interquartile range (IQR). A p-value < 0.05 was considered statistically significant. A paired t-test was used to compare parametric data for data comparing before and during the pandemic (e.g. comparing dysmenorrhoea experienced before and since the beginning of the pandemic). A Wilcoxon matched pairs rank test was used to compare non-parametric paired data.

## Results

### Patient Demographics

A total of 1031 women completed the survey. The participants demographic information is summarized in **Table 1**. The mean age of the women was 36.7 ± 6.6 years (range, 15–54 years). The mean self-reported body mass index (BMI) was 25.8 ± 5.5 kg/m^2^. (range, 16.6–65.5 kg/m^2^). 1000/97% of the women were of white ethnicity and 1007/98% were based in Ireland or the United Kingdom. 717/72% were married or cohabiting, 249/25% were single and 25/3% were separated or divorced. 593/58% stated that they had children and 74/7.2% were currently breastfeeding. 897/88% of women were working during the pandemic, 522/51% in the workplace and 375/37% from home. 326/59% of women who had children, home-schooled them when schools were closed and 369/66% had to provide childcare while also trying to work. 301/29% were healthcare workers (HCW); 175/17% Doctors, 57/6% nurses and 69/7% were other HCWs. 35/3.4% stated that they had contracted COVID-19 and tested positive, 63/6.1% had symptoms of COVID-19 but did not get tested.

**Table 1:** Participant demographics/medical history.

Demographics	
Age (mean ± SD), years	36.7 ± 6.6 (range, 15–54)
BMI (mean ± SD), kg/m^2^	25.8 ± 5.5 (range, 16.6–65.5)
Ethnicity	1000/97% White (White Irish, White British) 23/2% Asian 3/0.3% Black 5/0.5% Other
Location	862/84% Ireland 145/14% UK 24/2% other countries
Marital status	Single 249/25% Married 567/57% Cohabiting 150/15% Separated/Divorced 25/3% Widowed 1/0.1%
Occupation	301/29% Healthcare workers (HCW) 175/17% Doctors, 57/6% nurses, 69/7% other HCWs
Work status and location during pandemic	Full time in the workplace 392/38% Full time from home 311/30% Part time in the workplace 130/13% Part time from home 64/6% Maternity leave 47/5% Unemployed before pandemic 56/5% Made redundant/unemployed during pandemic 23/2%
Participants with children (yes/no)	Yes 593/58% No 435/42%
Currently breastfeeding	74/7.2%
Pre-existing medical conditions	Polycystic Ovary Syndrome 68/7% Excess unwanted hair 308/30% Hypothalamic amenorrhoea 3/0.3% Endometriosis 65/6% Premature ovarian insufficiency 22/2% Osteopenia/Osteoporosis 13/1% Acne 115/11% Thyroid disorder 91/9%
Covid-19 history “Did you have Covid-19?”	Yes and tested positive 35/3.4% Yes, had symptoms but did not get tested 63/6.1% No 870/83% No, but had contact with a confirmed case 63/6%

### Menstrual History

834/81% of women stated they usually had regular periods. 693/70% of those who had periods recorded them using an app, diary, smartphone or other recording method (**Table 2**). 747/72% of women were not using any form of contraception and 231/22% were using hormonal contraception (**Table 2**). The median cycle length was 28 days (27–30) with a 5 day bleed (4–6) and the minimum usual cycle length was 27 (25–28) days and the maximum usual cycle length was 30 days (28–32) (**Table 2**). 131/13% of women reported missing periods, 82/8% reported missing them occasionally and 49/5% often. 420/42% reported heavy periods and 416/42% painful periods (**Table 2**).

**Table 2:** Menstrual history and contraceptive use.

Parameter	Result
Contraception use	None—747/72% Combined contraceptive pill/patch—110/11% Intrauterine system—77/7% Progesterone only pill—31/3% Intrauterine device—24/2% Implant—13/1% Depot—2/0.2% Other—27/3% ( Barrier, tubal ligation, vasectomy)
Cycles recorded using app/diary/smartphone/other	Yes 693/70% No 297/30%
Regular periods under normal circumstances	Yes 834/81% No 163/16% N/A 32/3%
Median cycle length (days)	28 (27–30)
Median no. of days of bleeding	5 (4–6)
Minimum length of cycle (days) (median)	27 (25–28)
Maximum length of cycle (days) (median)	30 (28–32)
Missed periods	131/13% (occasionally 82/8%, often 49/5%)
Heavy periods	420/42%
Painful periods	416/42%

### Menstrual Cycle Changes During the COVID-19 Pandemic

441/46% of women who got periods reported an overall change in their menstrual cycle during the COVID-19 pandemic. See **Table 3** for menstrual cycle changes. 483/53% reported a worsening in premenstrual symptoms (PMS), whereas 60/7% felt that their PMS improved. The median cycle length was 28 days, similar to before the pandemic but with a significantly wider range (25–30) (p = 0.01). 255/29% noted a reduced cycle length and the median reduction was 3 days (2–6) and 28% reported a longer cycle with a median increase of 3 days (2–6). The median number of days of bleeding was 5 (4–6) and was unchanged compared to before the pandemic (p = 0.3). The minimum length of the cycle was 26 days (22–28), a median of 1 day shorter than before the pandemic (p < 0.0001). The maximum length of the cycle was 29 days (22–28), also a median of 1 day shorter than before the pandemic (p = 0.009).

**Table 3:** Menstrual cycle changes during the Covid-19 pandemic.

Change	Result, n/%	p-value
Overall change in menstrual cycle	Yes—441/46% No—523/54%	
Change in libido/sex drive	No change—429/42% Increased libido—131/13% Decreased libido—467/45%	
Change in premenstrual symptoms (PMS)	PMS unchanged—366/40% PMS better—60/7% PMS worse—483/53%	
Median cycle length (days)	28 (25-30) 255/29% reduced cycle length −3 days (2–6, range 1–55) 239/28% increased cycle length +3 days (2–6, range 1–103)	0.01
No. of days of bleeding (median, IQR)	5 (4–6)	P = 0.3
Minimum length of cycle (days) (median, IQR )	26 (22–28)	p < 0.0001
Maximum length of cycle (days) (median, IQR)	29 (28–32)	p = 0.009
Missed periods	−158/17% (+4%/27) (occasional—93/10%, often—65/7%) −72/9% new missed periods (occasional—56/7%, often—16/2%) -Median missed periods= 2 (1–3) -17/21% who ‘occasionally’ missed periods pre-pandemic missed periods ‘often’ during pandemic -40/31% who had missed periods previously had no missed periods during pandemic	p = 0.0003
Heavy periods	447/47% 18%/100—new heavy periods 15%/63—less heavy periods	P = 0.003
Painful periods	469/49% 173/30%—new painful periods 49/12%—previously painful periods improved	p < 0.0001
Conceived during pandemic	84/8%	

158/17% had missed periods during the pandemic, 4%/27 more than pre-pandemic (p = 0.0003). 72/9% reported new missed periods, of which 56/7% were “occasional” and 16/2% were “often.” The median number of missed periods was 2 (1–3). 17/21% who “occasionally” missed periods pre-pandemic missed periods “often” during pandemic. 40/31% of those who had missed periods previously had no missed periods during pandemic. 467/45% of women reported a decrease in their libido and 131/13% reported an increase in their libido. 447/47% of women reported heavy periods, 27/5% more than before the pandemic (p = 0.003). 469/49% reported painful periods, 53/7% more than before the pandemic (p < 0.0001). 173/30% reported new painful periods and 49/12% reported that previously painful periods improved during the pandemic.

### Lifestyle and Mental Health Change During the COVID-19 Pandemic

The overall median change in self-reported weight was an increase of 2 kg (0–4 kg) (p < 0.0001) (**Table 4**). 622/65% of women gained weight, and the median weight gain was 3 kg (2–5 kg) (**Table 4**). 158/16% lost weight, and the median weight loss was also 3 kg (2–5 kg) (**Table 4**). Women carried out on average 150 (40–270) min of exercise per week during the pandemic, 30 min (60–220) more than before the pandemic (p = 0.02) (**Table 4**). 517/50% of women felt that overall their diet was worse during the pandemic. 232/23% felt that their diet overall had improved (**Table 4**). 127/12% reported drinking excess alcohol compared to 72/7% before the pandemic (**Table 5**). 407/40% reported working more than before the pandemic and 169/16% were working less (**Table 4**).

**Table 4:** Changes in lifestyle during the COVID-19 pandemic.

Question	Result	P- value
Change in weight (n = 964) (median, IQR)	Median +2 kg (0–4) 184/19% no change in weight 158/16% lost weight, median −3 kg (2–5 kg) 622/65% gained weight, median +3 kg (2–5 kg)	<0.0001
Change in minutes of exercise/week (median, IQR)	+ 30 (40–270)	0.02
Type of exercise	Running—350/34% Yoga/pilates—300/29% HiiT—202/20% Strength training—209/20% Walking—193/19% Other—51/5% None—65/6%	
Diet	Overall diet is unchanged—281/27% Overall diet is better—232/23% Overall diet is worse—517/50%	
Change in work practices	No change—453/44% Working more—407/40% Working less—169/16%	

**Table 5:** Mental health symptoms.

	Before pandemic	During pandemic	P-value
Low mood	359/34%	519/50%	<0.0001
Anxiety	382/37%	514/50%	<0.0001
Poor sleep	341/33%	509/49%	<0.0001
Significant stress	268/26%	373/36%	<0.0001
Binge eating	236/23%	335/32%	<0.0001
Poor concentration	172/17%	360/35%	<0.0001
Loneliness	136/13%	299/29%	<0.0001
Poor appetite	64/6%	129/12%	<0.0001
Excess alcohol use	72/7%	127/12%	<0.0001
Illicit drug use	11/1%	7/1%	0.34

Women reported a significant increase in suffering from mental health symptoms (**Table 5**). 868/84% of women reported suffering from at least one symptom, including low mood (519/50%, p < 0.0001), anxiety (514/50%, p < 0.0001), poor sleep (509/49%, p < 0.0001), significant stress (373/36%, p < 0.0001), binge eating (373/36%, p < 0.0001), poor concentration (373/36%, p < 0.0001), loneliness (373/36%, p < 0.0001), poor appetite (373/36%, p < 0.0001) and excess alcohol use (373/36%, p < 0.0001). There was no change in illicit drug use (1%, p = 0.34). Women reported experiencing a number of stressors as outlined in **Table 6**, the most prevalent of which was work stress (499/48%), followed by difficulties accessing healthcare (254/25%) (**Table 6**).

**Table 6:** Stressors. “Have you had any of the following stressors over the course of the pandemic?”

Stressor	n/%
Work stress or change in employment status	499/48%
Difficulties accessing healthcare	254/25%
Change in financial situation	201/19%
Difficulties with home schooling	191/19%
Family illness or bereavement	169/16%
Change in living situation	156/15%
Family or partner conflict	170/16%
Difficulties providing or arranging childcare	99/10%

Women who reported experiencing one or more of low mood, anxiety, or significant stress were significantly more likely to report an overall change in their menstrual cycles since the beginning of the pandemic (50% versus 34%, p < 0.0001). These women with mental health symptoms were also more likely to report painful periods (54% versus 36%, p < 0.0001), worsening pre-menstrual symptoms (62% versus 32%, p < 0.0001), as well as decreased libido (51% versus 31%, p < 0.0001). 18% of women who experienced low mood, anxiety, and/or significant stress reported missing periods since the beginning of the pandemic, whereas 13% of those who did not experience these mental health symptoms reported missed periods, however this difference was not significant (p = 0.08). Those women who reported an overall change in their menstrual cycles were more likely to have reported poor sleep (41%) than those who did not report a change in their menstrual cycles (28%), p < 0.0001.

The final question of the study asked “do you have any other comments related to the impact of COVID-19 pandemic on your life?” 420/41% of respondents added a comment. Selections of the responses are provided in **Supplementary Table 2**. We labelled the comments as “positive,” “negative,” “mixed” or “neutral” depending on what impact was described by the respondents. 322/77% of comments described a negative impact of the pandemic on their lives, 41/10% described an overall positive impact of the pandemic, 27/6% commented that the pandemic had both positive and negative impacts on their lives, and 30/7% of comments were neutral.

## Discussion

This large, anonymous observational study has demonstrated that a large proportion of the female population have experienced reproductive health disturbance as a result of the COVID-19 pandemic. These disturbances are associated with a significant increase in suffering from mental health symptoms, as well as weight gain, longer working hours and an unhealthier diet. A minority of women have described improvement in their reproductive health and lifestyle over the course of the pandemic.

Women reported disturbances in their menstrual cycles that are known to be associated with psychological distress. Stress has an inhibitory effect on the hypothalamic pituitary gonadal axis (HPG). Stress and stress hormones inhibit GnRH release from the hypothalamus, and glucocorticoids inhibit luteinising hormone (LH) release and oestrogen and progesterone production by the ovary (18, 19). Stress regulates HPG axis through activation of hypothalamic sympathetic neural pathways that result in norepinephrine release in the ovary (20).

Functional hypothalamic amenorrhoea (FHA), chronic anovulation which is not due to an underlying organic cause, is associated with vigorous excess and an energy deficit, as well as stress, anxiety and mood disorders (7, 8, 21, 22). FHA has long-term health consequences including subfertility, osteoporosis, increased risk of cardiovascular disease and psychiatric disease (23). There was a significant increase in missed periods, likely as a result of psychological distress and an increase in the amount of exercise being carried out. Whether these missed periods will ultimately progress to chronic anovulation is as yet unknown. Women who missed periods occasionally before the pandemic reported missing them often during the pandemic. Given that many women gained weight and reported that their diet had worsened, this amenorrhoea is likely related to not only stress related amenorrhoea, but also overweight/obesity and worsening of PCOS symptoms, both known to be affected by incremental increases in weight (24, 25).

Over half of respondents reported worsening symptoms of pre-menstrual syndrome (PMS). Studies have demonstrated a higher prevalence of PMS among women with a high psychosocial stress level (15). PMS can have a significant impact on women’s health and is associated with impairment of activities of daily living and mental health disorders such as anxiety disorders, postnatal and perimenopausal depression (26). Almost half of women reported periods that were heavy and painful, a significant increase compared to before the pandemic. Again this is largely unsurprising as both have been shown to be associated with stress, psychological distress and low mood (12–15). 45% of women also reported a reduction in their libido. Hypoactive sexual desire disorder, when symptoms persist for over 6 months and are accompanied by distress, affects 6% to 13% of the European adult female population (27, 28). Self-reported reduced sexual desire has been shown to be associated with lower quality of life, poor self-esteem and hopelessness, as well as depression and anxiety (27).

Women reported no change in the median length of their cycle but there was a significantly wider range in the length of their cycle and a shortening of the minimum and maximum cycle length recorded. It is known that for those trying to conceive, shorter or longer menstrual cycles are less likely to be ovulatory and be followed by conception and are more likely to end in spontaneous abortion (29).

There was a significant increase in reported acute mental health suffering since the outbreak of the pandemic. Approximately half reported low mood and anxiety and approximately one third reported stress, binge eating, poor concentration and loneliness. Those who experienced low mood, anxiety and/or significant stress were more likely to report an overall change in their menstrual cycle, as well as worsening pre-menstrual symptoms, more painful periods, and reduced libido. Almost half of women reported poor sleep, and those who reported poor sleep were more likely to have experienced alteration in their menstrual cycle. It is known that sex hormones influence circadian rhythm, and vice versa (30, 31). PMS, which increased during the pandemic, is also associated with sleep disturbance (32). Sleep disturbance may actually affect fertility, as it has found to be more prevalent in those with infertility and those with diminished ovarian reserve (33).

Women also recorded lifestyle changes which may have impacted their menstrual cycles. There was an overall increase in the amount of exercise being undertaken, by half an hour per week. Despite this women gained weight, a median of 2 kg, likely because half of the women reported that their diet was worse and almost one third reported binge eating since the beginning of the pandemic. 40% of women were also working more during the pandemic, which would limit the time available for preparing healthy meals.

While a significant proportion of women described the negative impact of the pandemic on their menstrual cycle and lifestyle, there was a minority who described a positive effect. Some women had noted more regular periods, and periods that were less heavy and painful with less PMS. Some women reported an increase in their libido. There was an increase in average exercise per week, and 1 in 6 women lost weight. When they were invited to comment about the impact of the COVID-19 pandemic one in ten women described positive aspects of the pandemic; including a slower pace of life, less commuting and getting to spend more time with their families. Some women (6%) recognised both a positive and negative impact of the pandemic on their lives. It is as yet impossible to know if any positive effects will endure as the pandemic continues and progresses.

The major strength of this study is the large number of women surveyed, as well as the novel nature of the data. Another strength of the study is the fact that the majority (70%) of women were recording their menstrual cycle pattern using diary or smartphone app, therefore the menstrual cycle data is likely to be largely unbiased. In addition, the survey was anonymous, reducing the effect of social desirability bias, where people are more likely to report experiences that are considered to be socially acceptable.

There are several limitations to this observational study. The first is that the study recorded self-reported data, which is subject to bias. Self-reporting of menstrual cycle has been shown to have measurement error (34). However the majority of women in our study were recording their cycles using an app or a diary, reducing recall bias. Self-reported weight can also be inaccurate, however a study showed that when young people self-report weight they under-estimate by an average of only 0.4 kg (35), a modest under-estimate. In addition the mean BMI was 25.8 kg/m^2^, similar to previous large studies in women of reproductive age (36). Another potential limitation is that there may have been sampling bias, where those who opted to complete the survey were those who were more likely to have suffered menstrual disturbance. While we acknowledge that self-reported data collection is subject to bias, using digital surveys is a safe way to perform research during the pandemic, and given the anonymous nature of the study, the results have merit and provide a valuable insight into reproductive disturbances women have experienced as a result of the pandemic.

Another limitation is the population of women that completed the survey. The majority were of white ethnic background, so the data may not be representative of all women. The pandemic has disproportionately affected certain sectors of society, such as women of black, Asian or minority ethnic (BAME) background, who are more likely to be severely affected if they contract COVID-19 (37) and more likely to be from lower socio-economic backgrounds (38). 88% of respondents were in employment, which is higher than the European Union average for women of 66.5% (39). The average age was 36, and it is known younger women are more likely to develop hypothalamic amenorrhoea (40), so it is possible that a younger cohort of women may have worse symptoms. There was also a high number of healthcare workers surveyed, who may be more significantly affected by the pandemic due to enhanced workplace stressors and fear of exposure to COVID-19. Lastly, the reported mental health symptoms were intended only to provide a snapshot of how women were feeling subjectively, and are not an objective, qualitative measure of symptoms using validated mental health questionnaires. The main focus of this study was on the reproductive symptoms women were experiencing.

While it is clear from this study that women have suffered significant reproductive health disruption since the beginning of the pandemic, the medium and long term impacts of this are, as yet, unknown. This study captured the first approximately 6 months of the pandemic (February/March 2020 to September 2020). The longer term health implications are likely to also depend on the duration of the pandemic and other uncertainties such as the economic impact and the distribution of and access to COVID-19 vaccines. Future work should include continued multi-modal assessment at different intervals over the course of the pandemic, including ongoing validated assessment of mental health and measurements of BMI, hormone levels and ovulation.

## Data Availability Statement

The raw data supporting the conclusions of this article will be made available by the authors, without undue reservation.

## Ethics Statement

The studies involving human participants were reviewed and approved by St James’s and Tallaght University Hospitals REC. Written informed consent for participation was not required for this study in accordance with the national legislation and the institutional requirements.

## Author Contributions

LO is the primary investigator and completed data analysis and wrote the manuscript. NP wrote the manuscript and was involved in setting up and completing the study. LB wrote the manuscript and was involved in setting up and completing the study. All authors contributed to the article and approved the submitted version.

## Conflict of Interest

The authors declare that the research was conducted in the absence of any commercial or financial relationships that could be construed as a potential conflict of interest.
