# Supplementary material for: The Impact of the COVID-19 Pandemic on Women’s Reproductive Health
Source: Front Endocrinol (Lausanne). 2021 Mar 22;12:642755. doi: 10.3389/fendo.2021.642755 (PMC8030584; doi:10.3389/fendo.2021.642755)
Supplement: Supplementary file 1 [file Table_1.docx]

**Survey Questions**

1. What is your age?
2. What is your location?
3. What is your marital status?

- Single
- Married
- Cohabiting
- Separated/divorced
- Widowed

1. Do you have children?
2. Are you currently breastfeeding?
3. What is your occupation?
4. What is your current weight?
5. What is your height?
6. What is your ethnicity?

- White Irish
- White non Irish
- Asian/Asian Irish
- Black/Black Irish
- Irish traveller

1. Which of the following describes your current work practice?

- Full time in the workplace
- Part time in the workplace
- Full time working mainly from home
- Part time working mainly from home
- Furloughed
- Made redundant/unemployed
- On maternity leave
- Not working since before pandemic

1. Have you had a change in your work practice since the beginning of the COVID19 pandemic?

- Working more
- Working less
- No change

1. Were you home schooling when schools were closed?
2. Were you looking after your children while trying to work?
3. Did you have COVID19?

- Yes and tested positive
- Yes had symptoms but did not get tested
- No
- No, but I had contact with a confirmed case

1. Are you currently using any of the following contraceptives?

- Combined oral contraceptive pill
- Progesterone only pill
- Intrauterine system/coil (eg mirena/kyleena)
- Intrauterine Device (copper coil)
- Implant
- Depo injection
- None

1. Are/were you actively trying to get pregnant since the beginning of the COVID19 pandemic?
2. If yes have you conceived?
3. Do you usually get regular periods (before the COVID19 pandemic)?
4. Do you record your cycles using a diary/smartphone app/other method?
5. Overall have you noticed any change in your menstrual cycle over the course of the COVID19 pandemic?
6. Before the COVID19 pandemic, how many days of bleeding on average would you have during your period?
7. Since the beginning of the COVID19 pandemic, how many days of bleeding on average have you had during your period?
8. Before the COVID19 pandemic, how many days on average was your menstrual cycle?
9. Before the COVID19 pandemic, what was the minimum length of your menstrual cycle?
10. Before the COVID19 pandemic, what was the maximum length of your menstrual cycle?
11. Since the beginning of the COVID19 pandemic, how many days long on average has your menstrual cycle been?
12. Since the beginning of the COVID19 pandemic, what has the minimum length in days of your menstrual cycle been?
13. Since the beginning of the COVID19 pandemic, what has the maximum length in days of your menstrual cycle been?
14. Before the COVID19 pandemic would you say that you had heavy periods?
15. Since the beginning of the COVID19 pandemic, would you say that you have had heavy periods?
16. Before the COVID19 pandemic would you say that you had painful periods?
17. Since the beginning of the COVID19 pandemic, would you say that you have had painful periods?
18. Before the COVID19 pandemic would you say that you missed periods?

- Yes, often
- Yes, occasionally
- No
- N/A

1. Since the beginning of the COVID19 pandemic, would you say that you missed periods?

- Yes, often
- Yes, occasionally
- No
- N/A

1. How many periods would you say that you have missed?
2. Have you noticed any change in your premenstrual symptoms (PMS) over the course of the COVID19 pandemic? (e.g. Bloating, cramping, mood swings, irritability)

- PMS symptoms better
- PMS symptoms worse
- PMS symptoms unchanged
- N/A

1. Have you noticed any change in your libido/sex drive over the course of the COVID19 pandemic?

- Increased libido
- Decreased libido
- Libido unchanged

1. Do you have a diagnosis of PCOS (Polycystic Ovary Syndrome)?
2. Do you have any of the following that you know of?

- Hypothalamic amenorrhoea (athletic amenorrhoea)
- Endometriosis
- Early menopause/premature ovarian insufficiency
- Osteopaenia/Osteoporosis
- Acne
- Thyroid disorder
- None of the above

1. Do you have unwanted excess hair (eg facial hair, chest, back of thighs, lower abdomen)?
2. Please insert below the approximate change in your weight over the period of the pandemic
3. On an average week, how many minutes of exercise do you do in total?
4. By how much did your activity levels increase or decrease over the course of the COVID19 pandemic?
5. What type of exercise were you doing?

- Running
- Yoga/pilates
- HiiT
- Strength training
- Other
- None

1. Overall how do you think your diet over the course of the pandemic compares to your diet before the pandemic?

- Overall diet is better
- Overall diet is worse
- Diet is unchanged

1. Do you have a history of any of the following mental health issues?

- Anxiety
- Depression
- Eating disorder
- Alcohol excess
- None of above

1. Have you suffered from any of the following before the COVID19 pandemic?

- Low mood
- Poor appetite
- Binge eating
- Anxiety
- Poor sleep
- Poor concentration
- Loneliness
- Excess alcohol use
- Illicit drug use
- Significant stress
- None of the above

1. Have you suffered from any of the following during the COVID19 pandemic?

- Low mood
- Poor appetite
- Binge eating
- Anxiety
- Poor sleep
- Poor concentration
- Loneliness
- Excess alcohol use
- Illicit drug use
- Significant stress
- None of the above

1. Have you had any of the following stressors over the course of the pandemic?

- Change in financial situation
- Change in living situation
- Family illness/bereavement
- Family/partner conflict
- Work stress/change in employment status
- Difficulties with home-schooling
- Difficulty accessing healthcare
- Difficulties providing or arranging childcare
- None of the above

1. Do you have any other comments related to the impact of COVID19 pandemic on your life?
