# Supplementary material for: The Impact of the COVID-19 Pandemic on Women’s Reproductive Health
Source: Front Endocrinol (Lausanne). 2021 Mar 22;12:642755. doi: 10.3389/fendo.2021.642755 (PMC8030584; doi:10.3389/fendo.2021.642755)
Supplement: Supplementary file 2 [file Table_2.docx]

**Supplemental materials 2. Selection of responses to the question “Do you have any other comments related to the impact of COVID19 pandemic on your life?”**

| “Very stressful times for working parents. Especially front line workers who got no help with childcare etc from the government” |
| --- |
| “2020 so far has been THE worst year of my Life and still 3 months to go!” |
| “Very stressful we both are health care professionals and it was a very stressful time for us especially as my husband got Covid19 in work” |
| “It wasn’t until filling out this survey that I realised the actual impact the pandemic has had on me and how this has impacted my periods” |
| “I contracted Covid and still haven’t recovered 6 months later. It has devastated my life” |
| “Huge impact, children were home. My kids have special needs and they really struggled” |
| “My periods are so heavy. So heavy I thought I had a miscarriage in March. So heavy they have stained car seats. So heavy I can’t leave my house on day 2!!!! So heavy I had to go to the doctor. Clots and so much blood I can feel it rushing out of me!” |
| “1 very late period, more acne than usual, PMS symptoms that I never previously had” |
| “As someone who is single and looking for love, I worry about how best and sage to find a partner or romance in the future. Very conscious about my age and the likelihood of running out of time to have children” |
| “Constant low level anxiety at constant uncertainty” |
| “My last three periods have been sporadic and late. Before getting pregnant (and then the months after having the baby) my period was consistent and regular” |
| “Job difficulties, became unemployed and very little opportunities in my field” |
| “It’s been very tough to keep positive” |
| “Significant worries about my children and their mental health and physical wellbeing” |
| “Working from home and also minding young kids was very very stressful. I do t think I could do it again” |
| “Brought me closer to those who are important to me. Got to spend more time with them with less distractions” |
| “I found it very stressful and isolating working with a young family” |
| “I’m definitely much fitter as I have spent more time exercising which has overall improved my mood” |
| “I had a miscarriage and felt very isolated” |
